# The Study of Fingerprint Characteristics of Dayi Pu-Erh Tea Using a Fully Automatic HS-SPME/GC–MS and Combined Chemometrics Method

**DOI:** 10.1371/journal.pone.0116428

**Published:** 2014-12-31

**Authors:** Shidong Lv, Yuanshuang Wu, Jiangsheng Zhou, Ming Lian, Changwen Li, Yongquan Xu, Shunhang Liu, Chao Wang, Qingxiong Meng

**Affiliations:** 1 Faculty of Life Science and Technology, Kunming University of Science and Technology, Kunming, Yunnan, People's Republic of China; 2 Kunming Grain & Oil and Feed Product Quality Inspection Center, Kunming, Yunnan, People's Republic of China; 3 Yunnan Tasly Deepure Biology Tea Technology Limited Incorporation, Puer, Yunnan, People's Republic of China; ISA, Portugal

## Abstract

The quality of tea is presently evaluated by the sensory assessment of professional tea tasters, however, this approach is both inconsistent and inaccurate. A more standardized and efficient method is urgently needed to objectively evaluate tea quality. In this study, the chemical fingerprint of 7 different Dayi Pu-erh tea brands and 3 different Ya'an tea brands on the market were analyzed using fully automatic headspace solid-phase microextraction (HS-SPME) combined with gas chromatography-mass spectrometry (GC–MS). A total of 78 volatiles were separated, among 75 volatiles were identified by GC–MS in seven Dayi Pu-erh teas, and the major chemical components included methoxyphenolic compounds, hydrocarbons, and alcohol compounds, such as 1,2,3-trimethoxybenzene, 1,2,4-trimethoxybenzene, 2,6,10,14-tetramethyl-pentadecane, linalool and its oxides, α-terpineol, and phytol. The overlapping ratio of peaks (ORP) of the chromatogram in the seven Dayi Pu-erh tea samples was greater than 89.55%, whereas the ORP of Ya'an tea samples was less than 79.10%. The similarity and differences of the Dayi Pu-erh tea samples were also characterized using correlation coefficient similarity and principal component analysis (PCA). The results showed that the correlation coefficient of similarity of the seven Dayi Pu-erh tea samples was greater than 0.820 and was gathered in a specific area, which showed that samples from different brands were basically the same, despite have some slightly differences of chemical indexes was found. These results showed that the GC-MS fingerprint combined with the PCA approach can be used as an effective tool for the quality assessment and control of Pu-erh tea.

## Introduction

Tea (*Camellia sinensis*) is a popular drink consumed by two-thirds of the world's population. The major quality attributes of tea include appearance, the bottom of the leaf, aroma, color, and taste of the tea infusion. Consumer acceptability of tea depends predominately on its aroma and taste. Aroma and taste depend on the spatiotemporal variability of the production and production process, which in turn highly influences the chemical composition of tea and is critical in determining its quality [1], [2]. As a highly merchandised product, the evaluation and quality control of tea is very important and have been a popular research topic in recent years. Currently, the classification and evaluation of tea quality are mainly performed by trained experts who have developed a unique language to describe the various quality attributes of a tea infusion. However, this evaluation method is inconsistent in differentiating tea quality due to various factors, including individual variability and decreased sensitivity due to prolonged exposure, fatigue, stress, etc. [3], [4]. The taste and aroma of tea infusion are easily affected by many factors, such as production area, variety, cultivation techniques, picking seasons, manufacturing processes, and storage conditions [5], [6]. These factors make it very difficult for manufacturers to maintain consistent tea quality. With the recent development of the tea industry, determining a method to overcome these challenges has become more urgent than ever.

Chinese dark teas (CDTs) are post-fermented teas and represent one of the six major tea categories in China. CDTs mainly include Pu-erh tea (Yunnan Province), Ya'an dark tea (Sichuan Province), Liubao tea (Guangxi Province), and Heimao tea (Hunan Province) [7]. Pu-erh tea is the most representative CDT among these and has attracted global interest. Solid fermentation by microorganisms provides the tea's special characteristics including a brown-red color, mellow taste, stable flavor, and a clear, red-colored soup [8]. It has been demonstrated that Pu-erh tea has many potential health benefits, including prevention of oxidation, and antihypercholesterolemic, anti-hyperglycemic, anti-obesity and anti-diabetic effects [9], [10]. Pu-erh tea has been accepted and valued by an increasing number of consumers in the pursuit of a healthy lifestyle. Among the hundreds of tea manufacturers in Yunnan Province, Menghai Tea Factory is an industry leader in the production of Pu-erh tea. It mainly produces the “Dayi” brand, and its manufacturing process was listed in the Second National Nonmaterial Cultural Heritage Protection list. Because it is a special type of pile-fermentation tea, Pu-erh tea is not obviously different on the palate, but differs greatly in its aroma components. However, maintaining the consistency of product quality based on aroma components is extremely difficult. To solve this problem, a special production “tea blend” process was adopted by the Menghai Tea Factory. This process blended together various teas with different levels, production areas, seasons, varieties, production years, and degrees of fermentation. This process has the advantages of including complementary tea ingredients, improving product quality, and reducing the aromatic gap caused by different factors affecting Pu-erh tea [11]. In contrast, this process also leads to potential problems in quality control due to the lack of a reliable quantitative model for evaluation.

In recent years, chromatographic fingerprint techniques have been widely used in traditional Chinese medicines (TCM) for controlling product quality [12]–[14]. Compared with conventional methods, which focus on the determination of certain compounds, the fingerprint method can offer integral characterization of a complex system with quantifiable and reliable features [15]. Similar to TCMs, tea also contains a variety of constituents. This suggests that TCM fingerprint techniques can also be applied to study the chemical fingerprint of tea for its assessment and quality control.

HS-SPME is a unique pre-sampling method that requires no solvents or complicated apparatus. It integrates the extraction, concentration, and introduction in a single step. HS-SPME combined with GC–MS has been widely employed to determine volatile components in different tea samples, and the method has exhibited good repeatability, sensibility, and selectivity [16]–[18]. In contrast, most of the methods currently used are based on manual HS-SPME, which is labor-intensive with multiple samples. Fully automatic HS-SPME could shorten the total analysis time and achieve higher repeatability, thus improving productivity [19].

A high-performance liquid chromatograph (HPLC) fingerprint of Pu-erh tea and green tea has recently been reported [20], [21]. Additionally, paper spray mass spectrometry (PS-MS) was also used as a chemical fingerprinting methodology for assessing the overall quality of Bansha herbal tea [22]. Moreover, the chemometrics method has been applied to discriminate various fermented degrees [17] and different grades of tea [23], [24] based on the data obtained from the GC–MS. For its better repeatability and accuracy, wider ranges of measurements, and effective to both qualitative and quantitative analyses, GC-MS has been regarded as one of the most applicable and versatile technologies in the filed of analytical chemistry. However, to our knowledge, there have been no reports regarding the GC-MS fingerprint of tea.

In this work, the aroma components of 7 Dayi Pu-erh tea samples from different brands were analyzed by fully automatic HS-SPME coupled with GC–MS. The characteristic GC–MS fingerprints of these samples were compared by the fingerprint similarity evaluation system. The PCA analysis combined with chromatographic fingerprint data were used to further evaluate the similarities and differences among the tea samples. The objective of this study was to establish the characteristic fingerprint of Dayi Pu-erh tea and to provide a reliable and efficient methodology for quality control.

## Materials and Methods

### Ethics Statement

Our study did not require specific permissions for any locations or activities, and did not involve any endangered or protected species. Additionally, this study did not involve vertebrate or human ethics research. Our study area was located in the Yunnan Province of China (at east longitude 102°45′ to 103°00′, north latitude 24°42′ to 25°00′).

### Samples

Samples of seven different brands of Dayi Pu-erh tea were collected from the Menghai Tea Factory and numbered D1–D7 (Table 1). All types of Pu-erh tea were incorporated, including loose tea, cake tea, Tuo tea (a type of mini-packaged tea), and brick tea. In addition, three Ya'an dark tea samples (numbered A1–A3) were collected from Ya'an country in Sichuan Province and used as quality control. The manufacturing processes of these dark teas can be summarized as follows: fresh tea leaves are fixed by heat in a drum to inactivate the enzymes contained in the tea leaves. The tea leaves are subsequently dried and then combined with an adequate amount of water and piled into a windrow shape in the fermentation room for a period of time. During this process, the temperature of a windrow-shaped pile of tea leaves increases instantly at the beginning of fermentation and then decreases gradually to room temperature by the end of fermentation. Microorganisms accelerate the post fermentation process of dark teas by several weeks [25]. In this study, all dark teas were sealed and stored in a cool and dry place.

**Table 1:** Seven different brands of Dayi Pu-erh tea samples.

No.	Brands	Shape	Specifications
D1	7592	Seven sub-cake tea	357 g
D2	7572	Seven sub-cake tea	357 g
D3	Classic 7572	Sub-cake tea	150 g
D4	Chun Ping Pu-erh	Seven sub-cake tea	357 g
D5	V93	Tuo tea	100 g
D6	99 Fang Tea	Brick tea	81 g
D7	Chun Xiang Si Ji	Loose tea	80 g

### HS-SPME analysis

The fiber coatings (65 µm polydimethylsiloxane/divinylbenzene (PDMS/DVB)) were purchased from Supelco (Bellefonte, PA, USA). Prior to analyses, the fibers were conditioned 60 min at the manufacturer's recommended conditioning temperature.

The extraction procedure and chromatographic conditions were developed, validated and described in previous studies [26], [27]. All tea samples were ground into a fine powder and filtered with an 80-mesh filter. A ground tea sample (2.0 g) was weighed and placed in a 20 mL sealed headspace vial, and infused with 5 mL boiling water. After sampling, the HS-SPME procedures were performed using a Combi-PAL autosampler (Varian Pal Autosampler, Switzerland). The tea samples were continuously stirred at 250 rpm, and were extracted for 60 min at 80°C. After extraction, the fiber was immediately removed from the headspace and inserted into the GC injector for GC absorbance (250°C for 3.5 min) and analysis.

### GC-MS analysis

To analyze dark tea samples, a 7890A Agilent GC (Agilent Technologies, CA, USA) system equipped with a 5975C MSD detector (Agilent Technologies), autosampler (7683 B series, Agilent Technologies), and Chemstation software was used. An HP-5MS column (30 m×0.25 mm×0.25 µm film thickness) was used, and the gas carrier (helium at 99.999% of purity) was supplied at a constant rate of 1 mL/min. The injector temperature was 250°C, and the injection mode was splitless. The temperature program began at 50°C for 1 min and increased at a rate of 3°C/min until it reached 210°C (this temperature was held for 3 min), followed by temperature ramping at 15°C/min to a final temperature of 230°C. The mass spectrometer was operated under the electron impact (EI) mode at ionization energy of 70 eV. The aux temperature, MS source temperature, and MS quad temperature were set to 280°C, 230°C, and 150°C, respectively. A mass range of m/z 80–500 was scanned to confirm the retention times of target analytes and data were gathered in full scan mode. The solvent delay time was 2.8 min.

### Data analysis

Peaks were identified by searching the NIST08.L MS data library (a minimum match quality of 95% was used as the criterion for identification), and their retention indices (RI) were compared with published reports [28]–[32]. The relative percentages of the detected peaks were obtained by peak-area normalization, and all relative response factors were taken as one. To obtain the Kovats RI for each peak, 1 µL n-alkane mixture (C8–C40; Sigma-Aldrich, USA) was injected under the same GC-MS conditions. RI was calculated using:
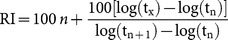
(1)where *t_x_* is the retention time, *n* and *n*+1 are respectively the number of carbon atoms in the alkanes eluting before and after the compound X.

Meanwhile, computer-aided similarity evaluation software, developed by the Research Center for Modernization of Chinese Medicines of the Central South University and based on Matlab 6.5, was specially coded to manage the GC-MS data. PCA was performed for the GC-MS data using the SIMCA package (Umetrics, Umea, Sweden), and differences between the samples were detected in the PCA score plots. Prior to runtime analysis of PCA, the data were processed by UV (unit variance) scaling. In addition, the overlapping ratio of peak (ORP) was utilized in this study. ORP indicates the correlation of two comparative samples according to their respective total number of peaks and common peaks [33]. This calculation method was based on a reference sample and the equation is as follows:
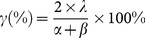
(2)


In this formula, γ indicates the overlapping ratio; λ represents the number of common peaks in two samples; α and β represent the number of total peaks in the reference sample and the compared sample, respectively.

In addtion, the calculation method of similarity coefficient of correlation based on the computer-aided similarity evaluation software was as follows:
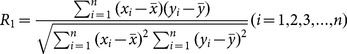
(3)where x_i_ and y_i_ are the ith measurements of x and y in the two TICs, and n is the number of the measurements in the TICs. 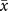 and 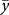 are the mean values of the n measurements in TICs x and y.

## Results and Discussion

### Repeatability and stability test

The repeatability was determined by performing five replicate HS-SPME/GC–MS experiments on the same D1 sample, under the same extraction conditions. The results showed that similarities of all D1 samples were more than 0.989, and the RSD values of the relative retention times and relative peak areas of volatile constituents were less than 0.2% and 8%, indicating a satisfactory repeatability of the developed method. With the same extraction conditions, the stability was determined by performing HS-SPME/GC–MS experiments on the same D1 sample at 0, 4, 8, 16, 24, 48 h, respectively. The results showed that similarities of all D1 samples were more than 0.976. The RSD values of the relative retention times and relative peak areas of volatile constituents were less than 0.3% and 7%, respectively. These results indicated that the method was reliable and applicable to the analysis of the chromatographic fingerprint of Pu-erh tea.

### Establishing the chromatographic fingerprints of Dayi Pu-erh teas

**Figure 1:**
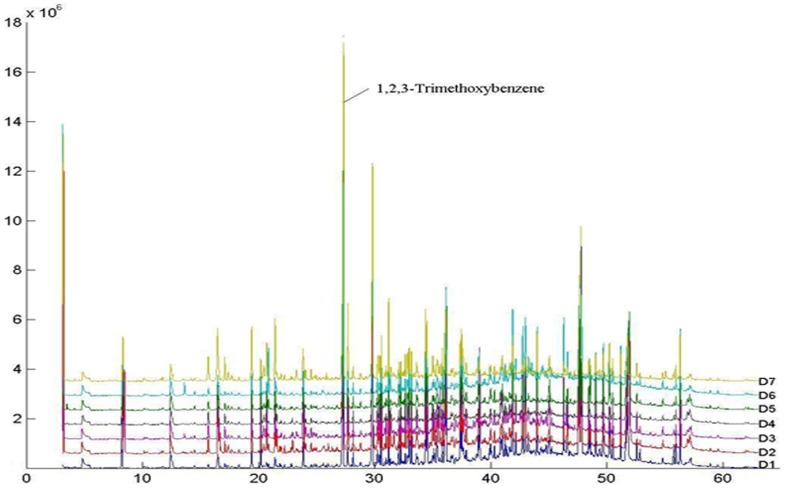
The overlapping plots of the GC-MS fingerprints in seven Dayi Pu-erh tea samples. D1–D7 represent different brands Dayi Pu-erh tea samples. 1,2,3-Trimethoxybenz in the figure was used as internal standard peak for calculating the relative retention value of other peaks.

In addition to the chromatographic peaks produced by column bleeding, a total of 78 peaks were determined in seven Dayi Pu-erh tea samples. A peak with 27.323 min was identified as 1,2,3-trimethoxybenzene. This peak existed in all the tea samples, had a high strength and was used as an internal standard peak for calculating the relative retention value of other peaks (the ratio of the retention time of the detected peak to the retention time of the internal standard peak). In addition, the D6 sample, which had the largest peak area and peak number, was selected as a reference sample. Using the relative retention value combined with the retention time and MS retrieval results of the chromatographic peak in each sample, 51 peaks were confirmed as common peaks. The relative standard deviation (RSD) values of the relative retention time of these 51 common peaks were less than 0.3%, which indicates that the retention time of each component was comparatively stable. Moreover, four other unique peaks existed in six Dayi Pu-erh tea samples, and six unique peaks existed in five tea samples. The 61 chromatographic peaks can be used as characteristic fingerprint peaks of the Dayi Pu-erh teas. Total ion chromatography (TIC) of volatile extracts from seven Dayi Pu-erh tea samples are shown in Fig. 1, and TIC of volatile extracts from A1–A3 (Ya'an dark tea samples) is shown in S1, S2, S3 Figs. (see supporting information). As shown in Fig. 1, all the samples had a high similarity in retention times while their peak abundances were different.

The ORP in seven Dayi Pu-erh tea samples were calculated with D6 as the reference sample and are listed in Table 2. The ORP in D1–D7 samples were all greater than 89.55%, while the ORP of the A1–A3 samples together were less than 79.10%. These results indicated that the samples D1–D7 shared different ORP with samples A1–A3, showing that their internal quality is distinctly different. This suggests that ORP can be further used to compare the differences between samples, using simple mathematics.

**Table 2:** The overlapping ratio of peaks and correlation coefficien similarity of 10 tea samples.

Sample no.	Overlapping ratio of peaks	Correlation coefficient
D1	91.04%	0.820
D2	91.97%	0.860
D3	91.73%	0.919
D4	89.55%	0.918
D5	90.23%	0.938
D6	100.00%	0.869
D7	90.51%	0.889
A1	76.81%	0.493
A2	77.55%	0.476
A3	79.10%	0.518

### Evaluating the chromatographic fingerprints of Dayi Pu-erh teas

**Figure 2:**
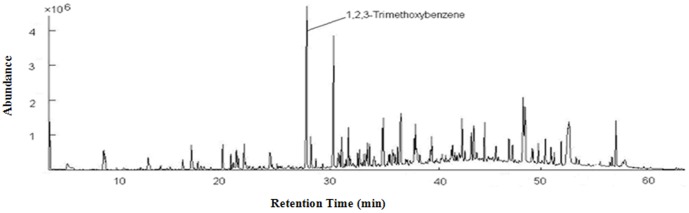
The mean fingerprint chromatograms of 7 Dayi Pu-erh tea samples, which represent common volatile components information of samples, and obtained by the fingerprint similarity evaluation system.

Fingerprints can reveal the similarities and differences (fuzziness) between different samples. The common chromatogram peaks existing in all samples represent their similarities, while non-common chromatogram peaks and the concentration differences of all peaks represent their differences. Therefore, it was crucial to build a characteristic fingerprint first, and then a correlation coefficient can be used to quantitatively describe the similarities and differences between the fingerprints [34]. In this work, a computer-aided similarity evaluation system was used to integrate the TIC of samples and to calculate the similarity degree between different dark tea samples. Additionally, this system can form a reference chromatogram based on the TIC of all samples, and quantify the difference between the sample fingerprints [35], [36]. Following area normalization and peak alignment, the correlation coefficients of each TIC to the reference chromatogram (Fig. 2) of all tea samples were calculated (Table 2). The results showed that the similarity values of the all Dayi Pu-erh tea samples were higher than 0.820, which suggested that gas chromatographic fingerprints obtained belonging to different brands Dayi Pu-erh tea samples were stable and basically the same. In contrast, the correlation coefficients of similarity of all A1–A3 samples were found to be less than 0.518. The different correlation coefficients of similarity of the Dayi Pu-erh and Ya'an teas indicated that the chromatographic fingerprints of these samples were significantly different.

There could be a few instrument parameter reaons that might produce some types of variation of TIC [37], such as drifts in retention time and even overlapping peaks. In our experiment, the correlation coefficient of the D1 sample was lower compared with the other Dayi Pu-erh teas, may be related to these factors. Dayi Pu-erh tea samples belonging to different brands showed a difference in aroma components and fingerprint fuzzification, which may be related to the selection of fresh raw tea leaves or to the blending and fermentation processes, but the fundamental similarities indicated that they were processed using the same or similar technologies and parameters and maintained a consistent quality.

### Digital profiling of aroma compounds in Dayi Pu-erh teas and Ya'an dark teas

In the TIC of the all dark tea samples, the chromatographic peaks were identified by NIST 08.L searching. In total, 78 major aroma compounds were separated, among 75 aroma compounds were identified in the Dayi Pu-erh teas (Table 3). In addition, a total of 80 major aroma compounds were identified and 3 aroma compounds were unknown in the three Ya'an dark teas (S1 Table, see supporting information). These results suggest that the composition of aroma compounds of Dayi Pu-erh and Ya'an teas is very complicated; the numbers of aroma compounds identified in the D1–D7 samples were 61, 63, 60, 61, 60, 67, and 64, respectively, and the number of aroma compounds identified in the A1–A3 samples were 66, 74, and 61, respectively.

**Table 3:** GC-MS analysis results of volatile compounds in 7 Dayi Pu-erh tea samples.

NO.	RI^a^	Compounds^b^	I.D.^c^	Relative percentage content (%)^d^						
				D1	D2	D3	D4	D5	D6	D7
1	957	Benzaldehyde	MS, RI	0.16	0.11	—	—	0.14	0.21	0.24
2	985	6-Methyl-5-hepten-2-one	MS, RI	—	—	—	—	—	—	0.11
3	989	2-Pentyl-furan	MS, RI	0.13	0.11	—	0.11	0.14	0.11	0.19
4	1030	2-Ethyl-1-hexanol	MS, RI	—	—	0.13	—	—	0.89	—
5	1048	1-Ethyl-1H-pyrrole-2-carboxaldehyde	MS, RI	0.16	—	0.15	0.11	0.24	0.38	0.17
6	1064	Acetophenone	MS, RI	—	—	—	—	—	0.16	—
7	1072	trans-Linalool oxide(furanoid)	MS, RI	0.31	0.63	0.61	0.80	0.51	0.65	1.53
8	1087	cis-Linalool oxide(furanoid)	MS, RI	0.90	1.99	1.94	2.06	1.39	1.50	3.25
9	1092	(E,E)-3,5-Octadien-2-one	MS, RI	—	0.16	—	—	—	0.16	—
10	1098	Linalool	MS, RI	0.22	0.40	0.43	0.52	0.31	0.30	0.96
11	1110	Phenylethyl alcohol	MS, RI	0.12	0.24	0.20	0.18	0.23	0.42	0.36
12	1149	1,2-Dimethoxybenzene	MS, RI	1.01	1.65	1.38	1.36	1.12	1.66	2.57
13	1169	cis-Linalool oxide(pyranoid)	MS, RI	0.12	0.38	0.64	0.47	0.32	0.35	0.52
14	1175	trans-Linalool oxide(pyranoid)	MS, RI	0.27	0.99	1.79	1.53	0.92	1.03	1.70
15	1178	Naphthalene	MS, RI	0.19	0.31	0.18	0.19	—	3.05	—
16	1188	α-Terpineol	MS, RI	0.67	1.40	1.84	1.64	1.15	1.28	2.72
17	1190	Methyl salicylate	MS, RI	0.14	0.70	0.39	0.55	0.31	0.66	0.34
18	1196	Safranal	MS, RI	0.17	0.19	—	—	0.16	0.23	0.10
19	1205	Decanal	MS, RI	0.16	0.25	0.14	0.15	0.13	0.19	0.21
20	1218	β-Cyclocitral	MS, RI	0.16	0.17	0.13	—	0.12	0.15	0.14
21	1228	Nerol	MS, RI	—	—	0.27	0.20	—	0.18	0.51
22	1241	3,4-Dimethoxytoluene	MS, RI	—	0.43	1.07	0.94	—	0.81	1.35
23	1256	Geraniol	MS, RI	0.15	0.29	0.33	0.25	0.18	0.26	0.38
24	1260	2-Methoxybenzyl alcohol	MS, RI	—	—	0.20	0.12	0.13	0.16	—
25	1265	3,5-Dimethoxytoluene	MS, RI	—	—	0.23	0.18	—	0.19	0.20
26	1283	1-Methoxy-4-(1-propenyl)-benzene	MS, RI	0.11	—	—	—	—	—	0.23
27	1287	2-Methyl-naphthalene	MS, RI	0.15	0.28	—	—	0.11	0.11	—
28	1294	2-Undecanone	MS, RI	—	0.13	—	—	—	0.11	0.12
29	1302	1-Methyl-naphthalene	MS, RI	0.21	0.24	—	0.15	—	0.25	—
30	1316	1,2,3-Trimethoxybenzene	MS, RI	9.35	17.69	16.24	13.69	15.02	14.14	21.01
31	1325	4-Ethyl-1,2-dimethoxy-benzene	MS, RI	0.80	1.84	2.36	1.66	1.45	1.60	2.89
32	1351	1,2-Dihydro-1,1,6-trimethyl-naphthalene	MS, RI	0.17	0.34	0.22	0.19	0.34	0.33	0.25
33	1375	1,2,4-Trimethoxybenzene	MS, RI	9.47	11.79	10.69	10.80	10.22	9.01	10.39
34	1384	Unknown-1^e^		0.61	0.66	0.74	0.86	0.62	0.67	0.78
35	1387	β-Guaiene	MS, RI	0.41	0.56	0.52	0.75	0.49	0.55	0.77
36	1397	cis-Jasmone	MS, RI	—	0.21	—	—	0.49	—	—
37	1400	Tetradecane	MS, RI	0.40	0.51	0.54	0.35	0.44	0.46	0.39
38	1406	1,2,3-Trimethoxy-5-methyl-benzene	MS, RI	0.90	1.01	3.41	2.60	2.07	2.49	3.03
39	1409	α-Cedrene	MS, RI	0.35	1.28	0.51	0.74	0.63	1.18	0.52
40	1417	β-Caryophyllene	MS	—	0.49	—	—	—	—	—
41	1428	α-Ionone	MS, RI	0.85	0.77	0.55	0.52	0.91	0.95	0.58
42	1432	Unknown-2^e^		0.95	0.92	0.58	0.59	1.24	1.25	0.56
43	1438	Dihydro-β-ionone	MS, RI	—	0.14	—	0.15	0.22	—	0.16
44	1442	1-Methoxy-naphthalene	MS	0.46	0.61	0.69	0.73	0.58	0.62	0.80
45	1447	2-Methoxy-naphthalene	MS	0.73	0.53	1.01	1.02	0.76	0.85	1.15
46	1449	1,2,3,4-Tetramethoxy benzene	MS, RI	1.34	1.59	1.26	1.13	1.57	1.17	1.07
47	1455	(E)-6,10-Dimethyl-5,9-undecadien-2-one	MS, RI	1.40	1.72	0.75	0.78	1.14	1.33	0.92
48	1483	4-(2,6,6-Trimethylcyclohexa-1,3-dienyl)- but-3-en-2-one	MS	1.02	0.92	2.82	2.18	2.30	2.54	2.52
49	1487	β-Ionone	MS, RI	3.90	3.46	2.05	2.26	3.45	3.14	2.24
50	1492	2-Tridecanone	MS, RI	—	1.41	—	—	—	—	—
51	1497	1,2-Dimethoxy-4-(1-propenyl)-benzene	MS, RI	0.63	—	0.89	0.96	0.52	0.55	0.89
52	1500	Pentadecane	MS, RI	0.54	0.74	0.63	0.36	0.35	0.50	0.29
53	1506	Dibenzofuran	MS, RI	1.57	—	0.75	0.71	0.74	—	0.68
54	1508	α-Farnesene	MS, RI	—	0.88	0.51	0.65	—	0.47	0.37
55	1519	Unknown-3^e^		0.94	0.98	0.92	1.03	1.36	0.93	0.95
56	1528	Dihydroactinidiolide	MS, RI	6.91	5.31	3.74	4.50	6.51	3.11	3.83
57	1554	Nerolidol	MS, RI	0.66	0.86	1.59	1.23	1.19	1.15	1.38
58	1572	Fluorene	MS, RI	0.65	0.68	0.76	0.54	0.72	0.54	0.61
59	1598	Cedrol	MS, RI	0.79	0.88	—	—	0.68	1.05	—
60	1600	Hexadecane	MS, RI	1.68	1.56	2.51	1.04	0.86	1.47	0.74
61	1653	α-Cadinol	MS, RI	1.01	0.65	1.02	1.15	0.88	1.04	0.75
62	1659	2,2′,5,5′-Tetramethyl-1,1′-biphenyl	MS	0.78	0.27	0.80	0.77	0.42	0.77	0.19
63	1664	2-Methyl-hexadecane	MS, RI	0.53	0.22	0.51	0.31	0.29	0.43	0.18
64	1700	Heptadecane	MS, RI	2.77	1.02	2.04	1.50	0.90	2.06	0.53
65	1706	2,6,10,14-Tetramethyl-pentadecane	MS, RI	4.61	1.57	3.02	3.12	1.45	2.92	0.68
66	1765	Anthracene	MS, RI	0.74	0.71	0.84	0.80	1.18	0.97	0.72
67	1800	Octadecane	MS, RI	2.00	0.49	1.67	0.75	0.65	1.98	0.48
68	1809	2,6,10,14-Tetramethyl-hexadecane	MS, RI	3.29	0.93	1.49	1.75	1.09	1.46	0.50
69	1840	Caffeine	MS	13.40	2.97	3.95	4.41	7.02	2.22	8.56
70	1846	6,10,14-Trimethyl-2-pentadecanone	MS, RI	2.85	2.67	2.15	2.55	3.70	2.77	1.41
71	1918	Farnesyl acetone	MS, RI	1.46	0.36	0.76	0.48	1.35	0.46	0.78
72	1927	Hexadecanoic acid methyl ester	MS, RI	1.87	0.41	0.54	0.69	0.60	0.48	0.72
73	1949	Isophytol	MS, RI	1.44	1.24	1.19	1.58	1.48	1.26	1.01
74	1975	Hexadecanoic acid	MS, RI	4.85	11.83	7.09	11.38	11.68	11.28	3.21
75	2000	Eicosane	MS, RI	0.53	0.15	0.26	0.21	0.26	0.23	0.22
76	2093	Methyl linoleate	MS, RI	0.67	—	0.19	0.25	—	—	0.24
77	2099	Methyl linolenate	MS, RI	1.35	0.27	0.37	0.45	0.40	0.30	0.50
78	2122	Phytol	MS, RI	2.88	2.87	2.85	4.30	2.16	1.88	1.65

a RI, retention indices as determined on HP-5MS column using the homologous series of n-alkanes.

b Compounds are listed in order of retention time.

c Method of identification: MS, identification by comparison with mass spectra; RI, identified by retention indices.

d Relative content, percent normalised peak areas; “—” not found or Relative percentage content <0.1%.

e mass spectral ions (relative abundance in %): unknown-1: m/z = 129 (100), 154 (98), 69 (82), 139 (80), 55 (73), 41 (64), 115 (39), 98 (35), 83 (23); unknown-2: m/z = 43 (100), 105 (87), 147 (82), 91 (66), 131 (44), 190 (40), 119 (37), 175 (36), 77 (33), 55 (29), 160 (17); unknown-3: m/z = 83 (100), 111 (45), 55 (33), 43 (14), 182 (13), 170 (10).

**Figure 3:**
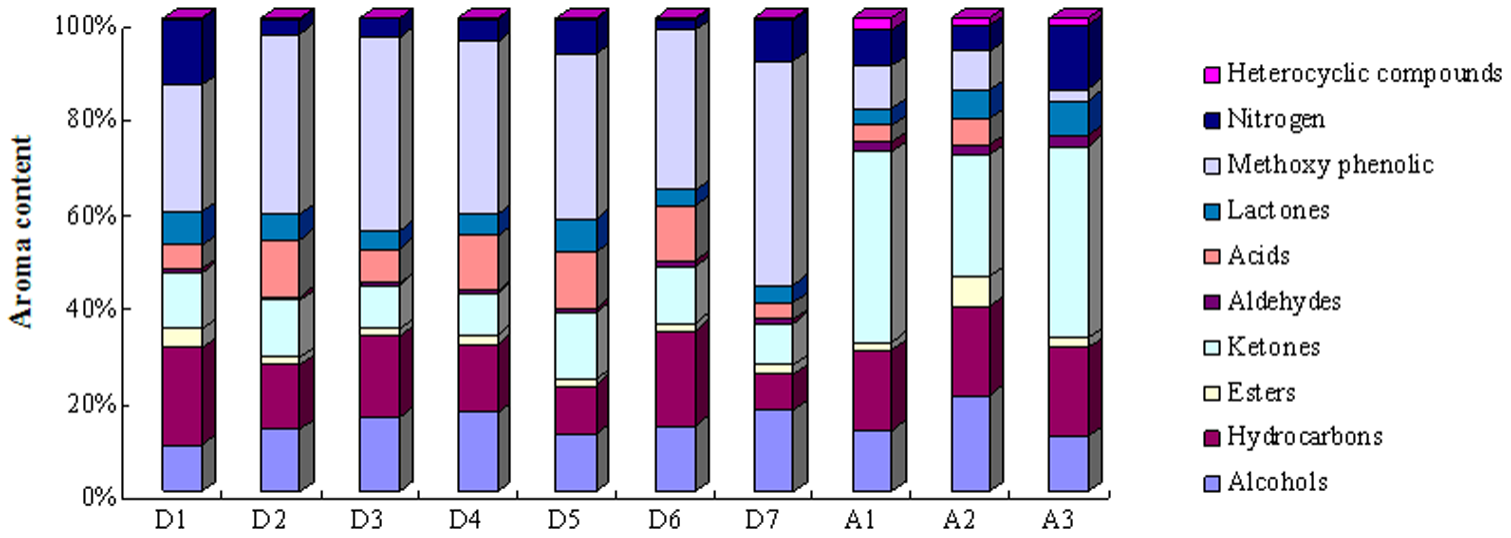
Comparison of aroma compounds between Dayi Pu-erh tea and Ya'an dark tea samples. Different colors represent different kinds of volatile components.

The aroma compounds identified by GC–MS in all the samples covered ten categories of organic chemical compounds. These compounds included methoxyphenolic compounds, alcohols, hydrocarbons, esters, acids, aldehydes, ketones, lactones, and nitrogen compounds, etc. The aroma compounds comparison result from the 10 samples is shown in Fig. 3. There were few differences in the aroma component content among the Dayi Pu-erh tea samples. Methoxyphenolic compounds (average content of 36.08%), hydrocarbons (14.54%), and alcohol compounds (13.58%) were the major aromatic components in the seven Dayi Pu-erh tea samples, while ketones (34.00%) and hydrocarbon compounds (17.38%) were the major aromatic components in the three Ya'an dark teas. Dayi Pu-erh teas contained significantly higher levels of methoxyphenolic compounds compared with Ya'an dark teas (*p*<0.05). Ya'an dark teas contained higher levels of ketones (*p*<0.05) than Dayi Pu-erh teas. There was no significant difference in the content with regard to alcohols and hydrocarbon compounds (*p*>0.05). The ester, aldehyde, lactone and nitrogen compound contents were relatively low in all tea samples.

The aroma composition undergoes a complicated transformation under the action of microorganisms during Pu-erh tea processing, which forms its many typical woody and stale components. The alcohols identified in the Dayi Pu-erh teas included linalool and its oxides, α-terpineol, α-cadinol, isophytol and phytol. These compounds have a typically floral and sweet scent. Different from green tea, black tea has a relatively high linalool content [38], [39]. The linalool content in Dayi Pu-erh teas is relatively low, but the content of linalool oxide is relatively high. This showed that linalool underwent a significant oxygenation process during the post-fermentation. In addition, Dayi Pu-erh teas have a high α-terpineol content; the formation of α-terpineol is mostly dependent on post fermentation with microbial activity [40], [41]. Phytol, with an elegant and sweet fragrance, and α-cadinol, with a woody fragrance, occupied a certain proportion among the samples, and contribute to the unique flavor of Dayi Pu-erh tea.

Among the hydrocarbon compounds identified in the Dayi Pu-erh tea samples, saturated hydrocarbons contributed little to the flavor, while unsaturated hydrocarbon compounds played an very important role in the tea's aroma [42]. However, there was a very low unsaturated hydrocarbon content in the Dayi Pu-erh teas. A total of 4 types of unsaturated hydrocarbons were detected and the average content of these compounds was only 1.80%. β-guaiene is an important volatile compound and endows Pu-erh tea with a unique woody scent [30]. Among the ester compounds, except for the methyl salicylate, which had a wintergreen oil herbal scent, other ester components were formed by higher fatty acids and lower alcohols. These ester componunds had poor volatility, and thus contributed little to the aroma of the tea [43]. Ketones occupied a certain proportion in the Dayi Pu-erh teas and mainly included 6,10,14-trimethyl-2-pentadecanone, α-ionone, β-ionone and €-6,10-dimethyl-5,9-undecadien-2-one. It was observed that α-ionone and β-ionone were the most important active odors of tea and contributed to the woody flavor, and β-ionone had a very low odor threshold (0.007 µg/L) [44]. Both of these compounds contribute enormously to the special flavor of Pu-erh tea.

It was obvious that methoxyphenolic compounds represent 36.08% (on average) of the total volatile constituents, were the major group in Dayi Pu-erh teas. Among these compounds, 1,2,3-trimethoxybenzene and 1,2,4-trimethoxybenzene were the most abundant components. Similar to our results, it has been reported that the amount of methoxyphenolic compounds and their derivatives coexist in Pu-erh tea samples at a high level [18], [45], [46]. Their amounts were found to increase remarkably from the sun-dried green tea to the Pu-erh tea product after the manufacturing process; these compounds were the key compounds in Pu-erh tea, effectively improving the roughage and aging odor of parched green tea and contributing to the stale and mellow aroma of Pu-erh tea [18]. Gas chromatography olfactometry (GC–O) and MS analysis have been used to characterize aroma components in Pu-erh tea [30]. The results showed that 1,2,3-trimethoxybenzene (17.16%) was the most abundant aroma component and played a vital role in the special stale flavor of Pu-erh tea. Furthermore, it has been reported that a number of methoxyphenolic compounds, such as 1,2,3-trimethoxybenzene and 1,2,4-trimethoxybenzene, were transformed by fungal methylate action from gallic acid (GA), and the methylation of phenolic hydroxyl groups by *Aspergillus niger* was also observed [25]. It can be deduced that the formation of these methoxyphenolic compounds has a close relationship with GA and microbial activity.

With respect to the rest of the identified components, hexadecanoic acid (3.21–11.83%) was found to be the major acid compound. Caffeine was also detected at a high level (2.22–13.40%) in Dayi Pu-erh teas. Both of these compounds were related to the taste of tea but contributed little to the aroma [47]. Dihydroactinidiolide (3.11–6.91%) was the only lactone compound detected in Dayi Pu-erh teas; its contribution to the aroma of Pu-erh tea needs to be confirmed with further studies.

### Principal component analysis of Dayi Pu-erh teas

**Figure 4:**
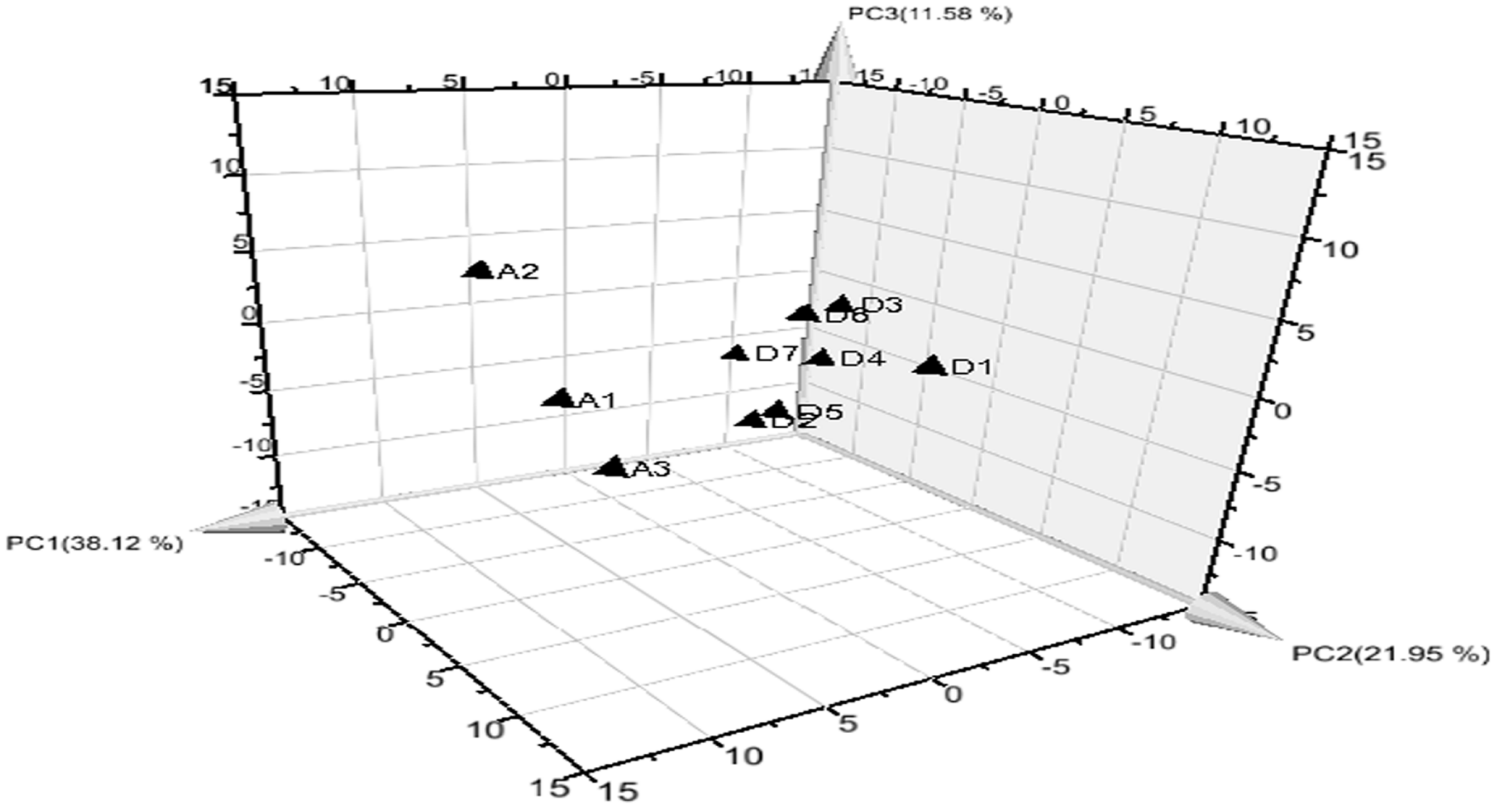
Score scatter plot of tea samples on the first three principal components. D1–D7 stand for the different samples in Table 1, and A1–A3 stand for different Ya'an dark teas. Samples were divided into two parts (left and right), showed the differences between them was obvious.

PCA is a linear transformation of the multiple variables into a lower dimensional space which retains the maximal amount of information about the variables [48], [49]. PCA could give a more visual comparison of the chromatograms, and the similarity analysis results could provide a more quantitative comparison of the samples. Entire compounds can be used to determine not only the absence or presence of desired markers or actives but also the complete set of ratios of all detectable analytes [50]. In this scenario, the relative content of each aroma compound (including the identified and unknown compounds shown in Table 1 and S1 Table) in the TIC was used for the data matrix. The data matrix used for the PCA was composed of a 10×98 data matrix (10 tea samples and 98 relative content values for aroma compounds). The results showed that four significant components can jointly account for 80.50% of the total variance (Fig. 4).

From the principal component score plots (shown in Fig. 4), there were no obvious differences among the Dayi Pu-erh teas, indicating that basic volatile compounds were similar. The PC1 values of A1–A3 were very different from those of the D1–D7 samples, indicating that there were obvious differences between these two groups of samples. In addition, the D1 and D7 samples deviated slightly from the central data values. This may be related to the methoxyphenolic and acid compound content values, which exhibited a greater difference in these two samples compared with other teas and also may relate to different taste characteristics. The results were consistent with the conclusions obtained in the correlation coefficient similarity comparison.

To establish a quality assessment system for Pu-erh tea and to provide a useful tool to evaluate the consistency of product quality, we tested different brands of Pu-erh tea samples obtained from the same manufacturers rather than different batches of the same brand. Therefore, the similarity values among Dayi Pu-erh tea samples in our results did not reach an ideal value (such as >0.95), and some differences were observed and reflected in the PCA results. However, it was notable that the samples had greater similarity values and were located relatively close together, which represented a better quality consistency of Pu-erh teas from the Menghai Tea Factory. Although the quality and consistency evaluation of Pu-erh tea is very important, the challenge is that the fingerprints can not see that which volatiles that make a significant contribution to the characteristic flavor of Pu-erh tea. Therefore, to some extent restricts the development and application of fingerprint methods. In the next study, more advanced instruments such as the GC-O should be used for the detection and identification of crucial and characteristic aromatic compounds, and then the relationships between these odor compounds can be explored. The number of samples used in this work was relatively small and the established model requires many samples to verify its accuracy. Therefore, additional dark teas from different production areas should be analyzed to establish the fingerprints and PCA model, thus providing more accurate information for the quality control of Pu-erh tea.

It is well known that the production process plays a key role in the formation of tea aroma components, which indirectly determines the quality of the tea. Further studies should be carried out to understand the balance (variability) of aroma compounds generated during the processing stages. These further studies will strengthen the aroma online fingerprint database and its applications in the quality control of Pu-erh tea and provide technical support for the production of high quality Pu-erh tea.

## Conclusions

In summary, this work reports, for the first time, the application of fingerprint analysis technology combined with chemometric methods to identify and evaluate the quality consistency of different brands of Dayi Pu-erh teas. A common fingerprint of Dayi Pu-erh teas was obtained; 51 common peaks coexisted in all seven of the studied samples, and their ORP values were all greater than 89.55%. A total of 75 volatile compounds were identified in seven Dayi Pu-erh teas, mainly including methoxyphenolic compounds, hydrocarbons, and alcohol compounds. The correlation coefficient of similarity and the PCA technique showed that Dayi Pu-erh teas had greater similarity values (≥0.820) and were clustered in specific domains, indicating that Dayi Pu-erh teas belonging to different brands were somewhat consistent. This method was also used to differentiate Ya'an dark teas from the Sichuan Province. The developed chromatographic fingerprint combined with the PCA technique can be used for the identification and consistency evaluation of Dayi Pu-erh teas. Such an approach is believed to be equally applicable to other teas.

## Supporting Information

S1 Fig **SPME/GC-MS chromatogram of volatile compounds in A1 tea samples.**
(TIFF)

S2 Fig **SPME/GC-MS chromatogram of volatile compounds in A2 tea samples.**
(TIFF)

S3 Fig **SPME/GC-MS chromatogram of volatile compounds in A3 tea samples.**
(TIFF)

S1 Table **GC-MS analysis results of volatile compounds in 3 Ya'an dark tea samples.**
(DOC)
