# Supplementary figures and images for: The Study of Fingerprint Characteristics of Dayi Pu-Erh Tea Using a Fully Automatic HS-SPME/GC–MS and Combined Chemometrics Method
Source: PLoS One. 2014 Dec 31;9(12):e116428. doi: 10.1371/journal.pone.0116428 (PMC4281233; doi:10.1371/journal.pone.0116428)

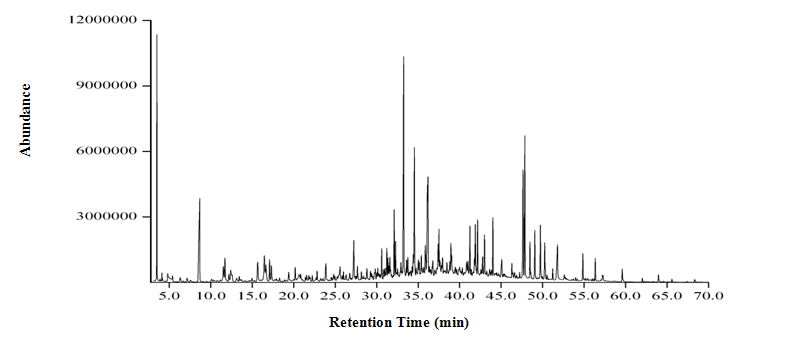

Supplement: S1 Fig — SPME/GC-MS chromatogram of volatile compounds in A1 tea samples. (TIFF) [file pone.0116428.s001.tiff]

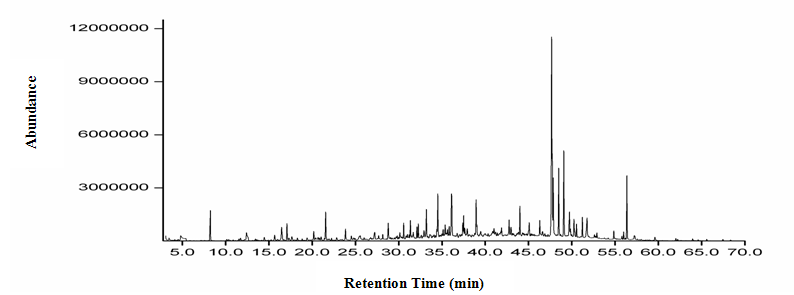

Supplement: S2 Fig — SPME/GC-MS chromatogram of volatile compounds in A2 tea samples. (TIFF) [file pone.0116428.s002.tiff]

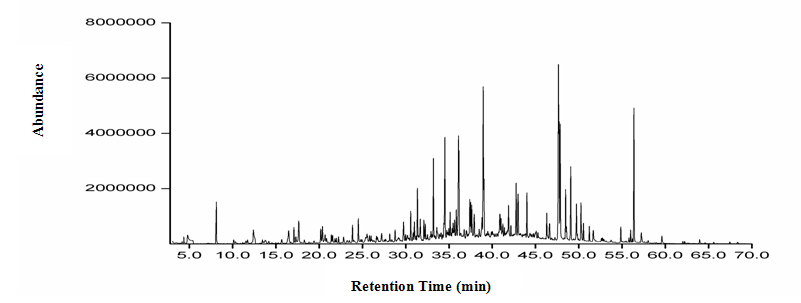

Supplement: S3 Fig — SPME/GC-MS chromatogram of volatile compounds in A3 tea samples. (TIFF) [file pone.0116428.s003.tiff]
