# Supplementary material for: The Study of Fingerprint Characteristics of Dayi Pu-Erh Tea Using a Fully Automatic HS-SPME/GC–MS and Combined Chemometrics Method
Source: PLoS One. 2014 Dec 31;9(12):e116428. doi: 10.1371/journal.pone.0116428 (PMC4281233; doi:10.1371/journal.pone.0116428)
Supplement: S1 Table — GC-MS analysis results of volatile compounds in 3 Ya'an dark tea samples. (DOC) [file pone.0116428.s004.doc]

**Table S1 GC-MS analysis results of volatile compounds in three Ya’an dark tea samples**

| No. | R.T. (Min.) | RI^a^ | I.D.^b^ | compounds | Relative percentage content (%)^d^ | | |
| --- | --- | --- | --- | --- | --- | --- | --- |
|  |  |  |  |  | A1 | A2 | A3 |
| 1 | 3.922 | — | MS | 2-Hexanone | — | 0.14 | — |
| 2 | 4.145 | — | MS | Hexanal | 0.31 | 0.14 | 0.26 |
| 3 | 6.311 | 861 | MS, RI | 1-Hexanol | 0.29 | — | — |
| 4 | 7.151 | 884 | MS, RI | 2-Heptanone | 0.29 | 0.45 | — |
| 5 | 10.105 | 957 | MS, RI | Benzaldehyde | 0.40 | 0.29 | 0.32 |
| 6 | 11.163 | 979 | MS, RI | 1-Octen-3-ol | 0.16 | 0.17 | — |
| 7 | 11.535 | 985 | MS, RI | 6-Methyl-5-hepten-2-one | 1.19 | 0.99 | 0.63 |
| 8 | 11.719 | 989 | MS, RI | 2-Pentyl-furan | 1.63 | 1.13 | 0.92 |
| 9 | 12.199 | 998 | MS, RI | cis-2-(2-Pentenyl)furan | 0.54 | 0.20 | 0.29 |
| 10 | 13.073 | 1018 | MS, RI | 1,2,3-Trimethyl-benzene | — | 0.15 | 0.14 |
| 11 | 13.441 | 1026 | MS, RI | D-Limonene | 0.30 | 0.38 | 0.32 |
| 12 | 13.715 | 1032 | MS, RI | Benzyl Alcohol | — | 0.22 | — |
| 13 | 15.265 | 1064 | MS, RI | Acetophenone | — | 0.17 | 0.13 |
| 14 | 15.675 | 1072 | MS, RI | trans-Linalool oxide(furanoid) | 2.14 | 1.84 | 0.28 |
| 15 | 16.468 | 1087 | MS, RI | cis-Linalool oxide(furanoid) | 2.29 | 3.51 | 0.78 |
| 16 | 16.729 | 1092 | MS, RI | 2-Nonanone | — | 0.27 | — |
| 17 | 17.110 | 1098 | MS, RI | Linalool | 1.31 | 3.18 | 0.73 |
| 18 | 17.620 | 1110 | MS, RI | Phenylethyl alcohol | — | 0.14 | 0.60 |
| 19 | 17.933 | 1117 | MS, RI | 1,2,3,4-Tetramethyl-benzene | — | 0.25 | — |
| 20 | 18.845 | 1137 | MS, RI | 2,5-Pyrrolidinedione, 1-ethyl- | — | 0.25 | 0.19 |
| 21 | 19.401 | 1149 | MS, RI | 1,2-Dimethoxybenzene | 0.62 | 0.50 | 0.24 |
| 22 | 20.403 | 1169 | MS, RI | cis-Linalool oxide(pyranoid) | — | 0.98 | 0.25 |
| 23 | 20.669 | 1175 | MS, RI | trans-Linalool oxide(pyranoid) | 0.51 | 1.20 | 0.28 |
| 24 | 20.849 | 1178 | MS, RI | Naphthalene | 0.27 | 0.46 | 0.34 |
| 25 | 21.427 | 1188 | MS, RI | α-Terpineol | 0.24 | 0.58 | 0.35 |
| 26 | 21.576 | 1190 | MS, RI | Methyl salicylate | 0.27 | 2.73 | 0.89 |
| 27 | 21.829 | 1196 | MS, RI | Safranal | 0.23 | 0.53 | 0.31 |
| 28 | 21.983 | 1200 | MS, RI | Dodecane | 0.36 | 0.24 | — |
| 29 | 22.240 | 1205 | MS, RI | Decanal | 0.38 | 0.36 | 0.57 |
| 30 | 22.823 | 1218 | MS, RI | β-Cyclocitral | 0.51 | 0.78 | 0.69 |
| 31 | 23.272 | 1228 | MS, RI | Nerol | 0.22 | — | — |
| 32 | 24.531 | 1256 | MS, RI | Geraniol | 0.24 | 0.62 | 0.35 |
| 33 | 25.255 | 1272 | MS, RI | Citral | 0.18 | — | — |
| 34 | 26.004 | 1287 | MS, RI | 2-Methyl-naphthalene | 0.42 | 0.46 | 0.31 |
| 35 | 26.334 | 1294 | MS, RI | 2-Undecanone | 0.44 | 0.33 | — |
| 36 | 26.753 | 1302 | MS, RI | 1-Methyl-naphthalene | 0.47 | — | 0.50 |
| 37 | 26.801 | 1304 | MS, RI | Isopropyl salicylate | — | 2.39 | — |
| 38 | 27.254 | 1316 | MS, RI | 1,2,3-Trimethoxybenzene | 2.82 | 4.17 | 1.43 |
| 39 | 27.687 | 1325 | MS, RI | 4-Ethyl-1,2-dimethoxy-benzene | 0.58 | 0.39 | — |
| 40 | 28.787 | 1351 | MS, RI | 1,2-Dihydro-1,1,6-trimethyl-naphthalene | 0.71 | 0.47 | 0.54 |
| 41 | 29.284 | 1362 | MS, RI | γ-Nonanolactone | — | 0.65 | 0.44 |
| 42 | 30.256 | 1384 |  | Unknown-1^c^ | — | 0.67 | 0.59 |
| 43 | 30.397 | 1387 | MS, RI | β-Guaiene | — | 0.58 | 0.37 |
| 44 | 30.860 | 1397 | MS, R | cis-Jasmone | 0.61 | 0.61 | 0.45 |
| 45 | 31.005 | 1400 | MS, R | Tetradecane | 0.49 | 0.90 | 0.82 |
| 46 | 31.219 | 1406 | MS, RI | 1,2,3-Trimethoxy-5-methyl-benzene | 1.33 | 0.66 | — |
| 47 | 31.361 | 1409 | MS, RI | α-Cedrene | 0.85 | 2.65 | 3.72 |
| 48 | 31.695 | 1417 | MS, R | β-Caryophyllene | 0.27 | 0.94 | 1.55 |
| 49 | 32.114 | 1428 | MS, RI | α-Ionone | 3.69 | 2.66 | 3.20 |
| 50 | 32.281 | 1432 |  | Unknown-2^c^ | 1.66 | 1.56 | 2.47 |
| 51 | 32.525 | 1438 | MS, RI | Dihydro-beta-ionone | 0.29 | — | 0.61 |
| 52 | 33.232 | 1455 | MS, RI | (E)-6,10-Dimethyl-5,9-undecadien-2-one | 13.55 | 5.54 | 11.67 |
| 53 | 33.750 | 1468 | MS, RI | 5-Methoxy-6,7-dimethyl-benzofuran | 1.64 | 0.80 | — |
| 54 | 34.427 | 1483 | MS | 4-(2,6,6-Trimethylcyclohexa-1,3-dienyl)but-3-en-2-one | 1.04 | 0.99 | 0.97 |
| 55 | 34.547 | 1487 | MS, RI | β-Ionone | 7.72 | 6.21 | 9.62 |
| 56 | 35.005 | 1497 | MS, RI | 1,2-Dimethoxy-4-(1-propenyl)-benzene | 0.92 | 0.43 | — |
| 57 | 35.133 | 1500 | MS, RI | Pentadecane | 0.65 | 0.64 | 0.87 |
| 58 | 35.257 | 1503 | MS, RI | Cuparene | — | 0.25 | — |
| 59 | 35.390 | 1506 | MS, RI | Dibenzofuran | 0.94 | 0.80 | 0.68 |
| 60 | 35.891 | 1519 |  | Unknown-3^c^ | 1.91 | 2.39 | 1.68 |
| 61 | 36.217 | 1528 | MS, RI | Dihydroactinidiolide | 3.63 | 5.04 | 6.57 |
| 62 | 37.634 | 1554 | MS, RI | Nerolidol | 1.05 | 0.62 | 1.15 |
| 63 | 37.972 | 1572 | MS, RI | Fluorene | 1.23 | 0.67 | 1.34 |
| 64 | 38.974 | 1598 | MS, RI | Cedrol | 1.26 | 3.96 | 3.72 |
| 65 | 39.017 | 1600 | MS, RI | Hexadecane | 0.80 | 0.50 | 1.09 |
| 66 | 40.837 | 1649 | MS, RI | Methyl jasmonate | 0.93 | 0.60 | — |
| 67 | 41.017 | 1653 | MS, RI | α-Cadinol | 0.8 | 0.73 | 0.66 |
| 68 | 41.261 | 1659 | MSI | 2,2',5,5'-Tetramethyl-1,1'-biphenyl | 2.61 | 3.68 | 0.77 |
| 69 | 41.402 | 1664 | MS, RI | 2-Methyl-hexadecane | 0.39 | 0.35 | 0.30 |
| 70 | 42.747 | 1700 | MS, RI | Heptadecane | 1.00 | 0.60 | 1.04 |
| 71 | 43.016 | 1706 | MS, RI | 2,6,10,14-Tetramethyl-pentadecane | 2.53 | 2.77 | 2.11 |
| 72 | 45.067 | 1765 | MS, RI | Anthracene | 1.84 | 0.30 | 0.41 |
| 73 | 46.283 | 1800 | MS, RI | Octadecane | 0.69 | 0.31 | 0.70 |
| 74 | 46.596 | 1809 | MS, RI | 2,6,10,14-Tetramethyl-hexadecane | 0.40 | 0.33 | 0.73 |
| 75 | 47.230 | 1828 | MS, RI | Isopropyl myristate | 0.24 | — | 0.23 |
| 76 | 47.748 | 1840 | MS | Caffeine | 7.31 | 4.96 | 12.71 |
| 77 | 47.855 | 1846 | MS, RI | 6,10,14-Trimethyl-2-pentadecanone | 7.85 | 5.16 | 9.57 |
| 78 | 50.244 | 1918 | MS, RI | Farnesylaceton | 2.04 | 0.94 | 1.99 |
| 79 | 50.531 | 1927 | MS, RI | Hexadecanoic acid, methyl ester | 0.30 | 0.38 | 0.65 |
| 80 | 51.208 | 1949 | MS, RI | Isophytol | 0.59 | 0.28 | 0.67 |
| 81 | 51.893 | 1975 | MS, RI | Hexadecanoic acid | 3.38 | 5.35 | — |
| 82 | 55.965 | 2099 | MS, RI | Methyl linolenate | — | 0.14 | 0.23 |
| 83 | 56.338 | 2122 | MS, RI | Phytol | 1.25 | 1.36 | 0.97 |

a RI, retention indices as determined on HP-5MS column using the homologous series of n-alkanes.

b Method of identiﬁcation: MS, identiﬁcation by comparison with mass spectra; RI, identiﬁed by retention indices.

c mass spectral ions (relative abundance in %): unknown-1: m/z = 129 (100), 154 (98), 69 (82), 139 (80), 55 (73), 41 (64), 115 (39), 98 (35), 83 (23); unknown-2: m/z = 43 (100), 105 (87), 147 (82), 91 (66), 131 (44), 190 (40), 119 (37), 175 (36), 77 (33) , 55 (29), 160 (17); unknown-3: m/z = 83 (100), 111 (45), 55 (33), 43 (14), 182 (13), 170 (10).

d Relative content, percent normalised peak
